# Uncovering inequality through multifractality of land prices: 1912 and contemporary Kyoto

**DOI:** 10.1371/journal.pone.0196737

**Published:** 2018-04-30

**Authors:** Hadrien Salat, Roberto Murcio, Keiji Yano, Elsa Arcaute

**Affiliations:** 1 Centre for Advanced Spatial Analysis, University College London, London, United Kingdom; 2 Consumer Research Data Centre, University College London, London, United Kingdom; 3 Department of Geography, Ritsumeikan University, Kyoto, Japan; CNRS, FRANCE

## Abstract

Multifractal analysis offers a number of advantages to measure spatial economic segregation and inequality, as it is free of categories and boundaries definition problems and is insensitive to some shape-preserving changes in the variable distribution. We use two datasets describing Kyoto land prices in 1912 and 2012 and derive city models from this data to show that multifractal analysis is suitable to describe the heterogeneity of land prices. We found in particular a sharp decrease in multifractality, characteristic of homogenisation, between older Kyoto and present Kyoto, and similarities both between present Kyoto and present London, and between Kyoto and Manhattan as they were a century ago. In addition, we enlighten the preponderance of spatial distribution over variable distribution in shaping the multifractal spectrum. The results were tested against the classical segregation and inequality indicators, and found to offer an improvement over those.

## Introduction

Reardon et al. [1] pointed out the necessity for new spatial economic segregation and inequality measures insensitive to the choice of category thresholds, boundary definitions, and shape-preserving changes in the variable distribution. Despite improving the situation, we have identified that the new measures proposed in the same article still face boundary definition biases (a common issue in spatial statistics usually referred to as the Modifiable Areal Unit Problem, or MAUP) and are unsatisfactorily insensitive to all changes in the variable distribution, including non shape-preserving ones. Multifractal analysis could offer a good alternative, free of all the aforementioned problems, for sets obeying a number of scaling conditions.

It is well known that scaling often emerges in self-organized complex systems, and the fractality of urban structures in particular is well documented [2–4]. It has been argued that a single fractal exponent may not be sufficient to fully characterize urban systems, and that land prices could be better described by multifractals [5, 6]. The purpose of this article is to make use of two highly detailed land-prices datasets for Kyoto spanning over a hundred years [7–10], and of urban simulated models based on this data to confirm that multifractals are indeed a relevant description of the spatial heterogeneity of land price measures in this case, and to set a basis for the interpretion of multifractal spectra in terms of inequality and segregation. The information obtained from multifractal analysis is compared in detail to the information resulting from classical inequality and segregation measures (such as Gini, Theil, Neighbourhood Sorting, Ordinal Information Theory and Ordinal Variation Ratio indices). We show that the information from classical tools is recovered using multifractal analysis, and that some additional information is gained.

The main factual results are a sharp decrease in multifractality for present Kyoto compared to Taisho era Kyoto (a trend for modern cities in line with other studies [11, 12]), and a striking similarity both between present Kyoto and present London, and between Kyoto and Manhattan as they were a century ago. From the way the multifractal spectrum shrank, we can infer an increase in local homogeneity for the modern city and hints at densification. Furthermore, we show that for this data the resulting spectra are primarily dictated by the shape of the spatial distribution rather that the shape of the price distribution, a characteristic highly sought after by Reardon et al. [1].

We first introduce the methodology and its interpretation for the analysis of inequality, together with a recap of the technical choices made to suit the methods to the current data. We then present the main results, first for Taisho era Kyoto and its associated simulated models, second for present Kyoto and its associated models, and third we compare the two timestamps between themselves and with data for present Manhattan and London. The final section is a discussion mainly focused on the comparison between the multifractal methodology and classical inequality tools.

## Methodology

We first present a formal definition of multifractality, then a heuristic interpretation and an illustration of the main variables. We explain next how the methodology was adapted to suit a real estate context, and how the data was prepared to mitigate its imperfections. The technical details on the actual computation can be found in Appendix A1 in S1 Appendix.

### Multifractal measures

Multifractal theory [13–15] can be used to study the heterogeneity and irregularity of measures defined on sets that are too irregular for classical geometric tools, when those measures present two separate scaling properties. Consider a measure *μ* defined on a set *A*, it is required that

1. locally around any point *x* of the set *A*, the measure is scaling with a local exponent *α*_*x*_;
2. the set formed by all points around which the measure scales with the same local exponent *α*_*x*_ is a fractal set of dimension *f*(*α*_*x*_).

There are several more or less equivalent definitions of “fractal” sets. Here, we will consider *fractal* any set for which a box-counting dimension can be computed (regardless of whether said dimension is a fraction or not, or whether the set is self-similar in a strict sense or not). The curve *f*(*α*) against *α* is called the *multifractal spectrum*. It gives, roughly speaking, the “fractal dimension” *f*(*α*) of sets where the measure scales locally with the same exponent *α*.

Formally, denoting *μ*_*r*_(*x*) the measure in a ball of radius *r* around *x* and *N*(*α*) the number of times an *α*_*x*_ falls inside the interval [*α*, *α* + *dα*], the two scaling properties mentioned above can then be written

1. $\mu_{r}\left(x\right)\propto r^{\alpha_{x}}$ for some *α*_*x*_ around any *x* ∈ *A* when *r* is small enough;
2. *N*(*α*_*x*_) ∝ *r*^−*f*(*α*_*x*_)^, for some function *f* and any *x* ∈ *A*.

The goal is to find all the *α*_*x*_ and *f*(*α*_*x*_) values in the system. To this end, we will use the classical moment method [16–18], and in particular its multiplier variant (see [19, 20]). The data is divided into a grid whose squares are numbered. Denoting *μ*_*i*_(*r*) the measure of a Moore neighbourhood of radius *r* around square *i*, the quantity *Z*(*q*, *r*) is defined for any real number *q* by

$$\begin{array}{c} \begin{array}{cc} Z\left(q,r\right)≔\sum_{i}\mu_{i}{\left(r\right)}^{q} & \propto \sum_{i}r^{\alpha_{i}q}\\ & \propto \sum_{\alpha }N\left(\alpha \right)r^{\alpha q}\\ & \propto \sum_{\alpha }r^{\alpha q-f(\alpha )}. \end{array} \end{array}$$ (1)

For each *q*, when *r* → 0, only the value of *α* that minimizes *αq* − *f*(*α*) makes a significant contribution to the sum, so that the *α* and *f*(*α*) values can be deduced from a Legendre transform of the quantity

$$\begin{array}{c} \tau \left(q\right)≔\alpha \left(q\right)q-f\left(\alpha \left(q\right)\right)\approx \lim_{r\to 0}\frac{\log(Z(q,r))}{\log(r)}. \end{array}$$ (2)

Going through all *q* in R yields all *α* and *f*(*α*) values. When using the multiplier and gliding box variant, boxes of some minimal size *r*_0_ and some other size *r*_*k*_ are glided along the grid. The *τ*(*q*) and *α*(*q*) values are then directly computed from the expressions

$$\begin{array}{c} \tau \left(q\right)+d\approx -\frac{\log(1/N\sum_{i}M_{i}^{q})}{\log(r_{k}/r_{0})}; \end{array}$$ (3)

$$\begin{array}{c} \alpha \left(q\right)\approx -\frac{\sum_{i}M_{i}^{q}\log\left(M_{i}\right)}{\sum_{i}M_{i}^{q}\log\left(r_{k}/r_{0}\right)}, \end{array}$$ (4)

where *i* is the grid cell corresponding to the centre of the box while it is glided, *M*_*i*_: = *μ*_*i*_(*r*_0_)/*μ*_*i*_(*r*_*k*_), *N* is the number of non zero values of *M*_*i*_, and *d* is the dimension of the physical support *A*. In a sense, the multiplier method infers the underlying power law from the way the measure grows between two scales. A very detailed explanation of its working principles can be found in [19].

### Spectrum interpretation

In order to get a heuristic idea of what the *α* values represent, assume that we want to study the multifractality of a non-negative signal *ϕ* defined on a one-dimensional space, say R. We can create a measure *μ* from this signal by defining for any a<b∈R

$$\begin{array}{c} \mu \left(\left[a,b\right)\right)=\int_{a}^{b}\phi \left(x\right)dx. \end{array}$$ (5)

**Fig 1:**
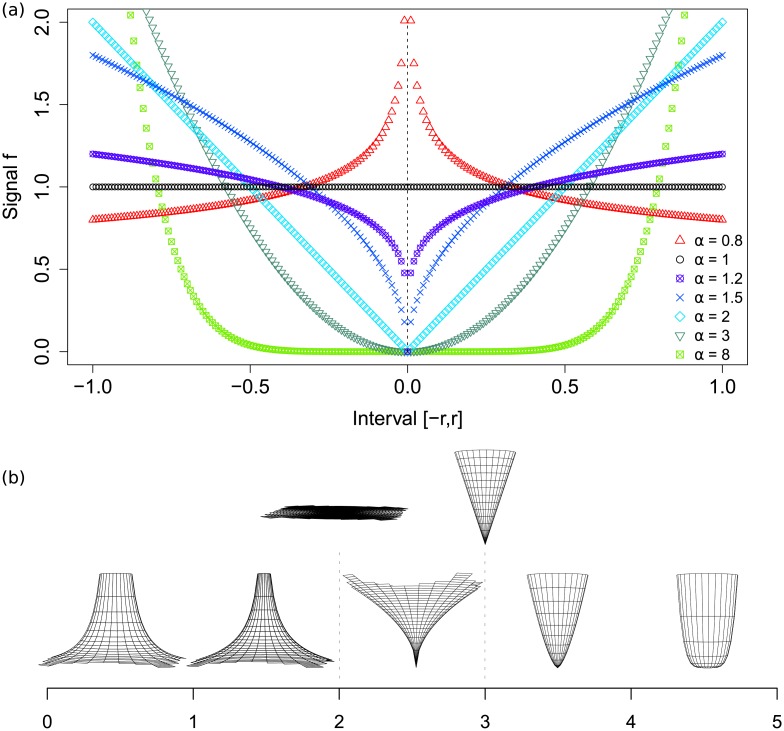
Meaning of the *α* values. (a) Idealized local behaviour of a one-dimensional signal around a point of strength *α*. (b) Idealized behaviour of a two-dimensional signal around a point whose strength *α* is indicated on the scale below.

In particular, the first scaling rule *μ*([0, *r*)) ∝ *r*^*α*^ translates into *ϕ*(*x*) ∝ *x*^*α*−1^ on the interval [0, *r*). Assuming there is a reflectional symmetry around 0, this formalization allows us to illustrate the typical limiting behaviour of a multifractal signal around a point corresponding to some particular *α* when *r* → 0 (see Fig 1a). Note that choosing to place the point in 0 and the vertical scaling are both arbitrary in this example.

If we were to consider instead a perfectly isotropic two-dimensional signal, then the cross-cut along any particular direction would be the same as the curves above at the condition of adding 1 to the corresponding *α*. A representation of such a signal for particular *α* values can be found in Fig 1b. The real signal does not have to be isotropic, for example “bumps” on one side of a circle of radius *r*_0_ can be compensated by “holes” on the other side of the same circle.

It must be emphasized that *α* values represent the rate at which a signal, and more generally any measure considered, grows around a point in contrast to the actual height of its point value. However, the *α* values are not completely invariant through vertical translation of the signal. Assume that a signal is of the form *r*^*β*^ + *k* for some *β* and a real constant *k* > 0. Then, once integrated, the measure will be either of the form *r*^*α*^ + *k* * *r* or *r*^*α*^ + *k* * *r*^2^ for one-dimensional or two-dimensional signals respectively. The noise introduced by *k* is therefore negligible for *α* values below 1 or 2 (resp.) when *r* → 0, while the measure is negligible compared to the noise for *α* values greater than 1 or 2 (resp.) when *r* → 0. As a matter of fact, the standard moment method applied to real data sometimes seems to fail for *α* values greater than 1 or 2 (e.g. [5, 11, 20]).

The basic moment method may fail for other reasons, such as the presence of underlying large-scale structures in the data. This has been extensively discussed in [21, 22]. In contrast, the multiplier method ignores the potential noise and large-scale biases by inferring a true local power-law around each point from the way the signal grows around it at two different scales. In particular, for *α* values greater than 1 or 2, it extends the local behaviour of the measure as if it would reach 0 in 0 despite the noise *k*. In a sense, the multiplier process can be thought of as a way of “calculating the best multifractal fit” for the measure.

**Fig 2:**
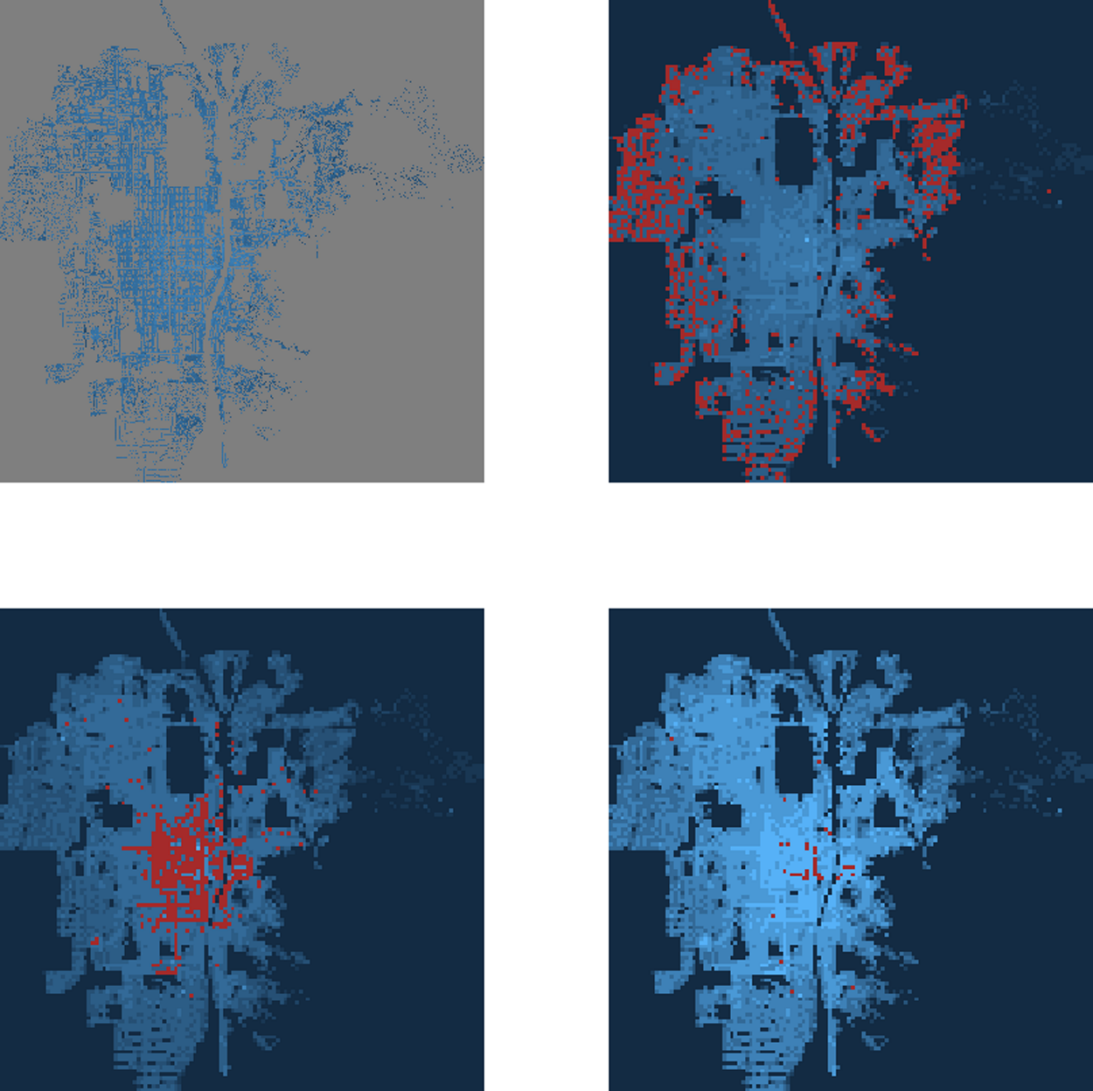
Meaning of the *f*(*α*) values. Top left: real price distribution for Kyoto in 1912 in a log-scale. The other figures represent the corresponding non-rigorous *α* distribution with: top right: *α* = 3 highlighted in red; bottom left: *α* = 6 highlighted in red; and bottom right: *α* ≥ 7 highlighted in red. For each *α* value, the dimension *f*(*α*) is akin to the box-counting dimension of the highlighted zone.

The *f*(*α*) values may mean slightly different things depending on how they have been defined. Here, they are assimilated to the box-counting dimension of the set formed by all squares in a grid whose measure shares the same strength *α*. This is illustrated in Fig 2. On the top left, the land price distribution for Kyoto in 1912 is represented in a blue continuous logarithmic scale applied over a 512x512 grid. In the three other images, the 4x4 squares corresponding respectively to a strength of *α* = 3, *α* = 6, and *α* ≥ 7 (counting clockwise) are highlighted in red. The values *f*(*α* = 3), *f*(*α* = 6), and *f*(*α* ≥ 7) are the box-counting dimension of each highlighted zone. Those values were obtained by fitting directly power laws around the centre of neighbourhoods of size 4x4. The true *α* values require to take the limit when the neighbourhoods size approaches 0. The ones shown here are for illustrative purposes only.

Since lower and higher *α* values mean sharper growth, while values around 1 for one-dimensional measures and around 2 for two-dimensional measures are a sign of local homogeneity (with only isolated singularities), some non-intuitive insights on segregation can be deduced from the repartition of the *α* values. Indeed, if the *α* distribution is narrow and centred around 1 or 2, while the spread of the measure is high, it means that the prices are clustered into groups of locally similar *α* values (for which the *f*(*α*) dimension is close to 2), while the lower and higher *α* values represent the sharper edges of the clusters (for which the *f*(*α*) dimension is closer to 1). In contrast, if both the measure spread and the *α* distribution are narrow, then segregation is low.

In particular, it has been evidenced by Murcio et al. [11] that there is a loss of multifractality over time in modern cities, which translates into a shrinking of the spread of *α* values. The remark above encourages to pay extra attention to the symmetries of the shrinking, rather than to its extent alone.

### Application to measuring land ownership inequality and datasets

As the input for the multifractal moment methods needs to be a mathematical measure, continuous densities cannot be used directly, and a suitable base unit had to be chosen to count the measure. For real estate, several units inferring different interpretations can be considered.

To describe the spatial distribution of land value, the unit could be each square meter of land. In order to describe property value instead of land value, the mathematical definition of a measure imposes to consider the total housing value carried by each unit of land. In the case of a multi-storey building, one should therefore count the total available floorspace per unit of land, rather than the average value on that unit of land. For example, if a square meter of land *l*_1_ is underneath a 15 stories building at an average sqm price *p*_1_ and another square meter of land *l*_2_ is underneath a one storey house at a sqm price *p*_2_, then the total property value carried by *l*_1_ is 15 × *p*_1_, and the total property value carried by *l*_2_ is *p*_2_. Considering *p*_1_ instead of 15 × *p*_1_ for *l*_1_ would underestimate the real impact of price *p*_1_ in the city compared to price *p*_2_.

To study housing inequality, each accommodation can be considered as a unit of its own, independently of its size. The results would then give information on the affordability of suitable housing assets and on the spatial repartition of each price category. Adopting the point of view of policy makers, one could choose a set number of people as the base unit and try to calculate the cost of accommodating that number of people.

Unfortunately, the data available may have the final word on deciding the unit. For early 20th century Kyoto, we have based our work on the digitalization of a cadastral map first published in 1912 (first year of Taisho era) by Yano et al. [8–10]. At the smallest level, all land is divided into 64486 lots registered to a single owner for which price, category, and area are known. We used the total price of each lot marked as residential as the base unit for the analysis. Assuming that the land lots are not meant to be divided, it represents accurately all existing residential land assets in the city. We could have considered lots of categories other than residential to represent assets for future development of the city, but given that the other datasets do not contain non-residential values, we focus exclusively on built land to be consistent. For the record, non-residential land represents 18% of all unique lots. The dataset is available in S1 Dataset.

**Fig 3:**
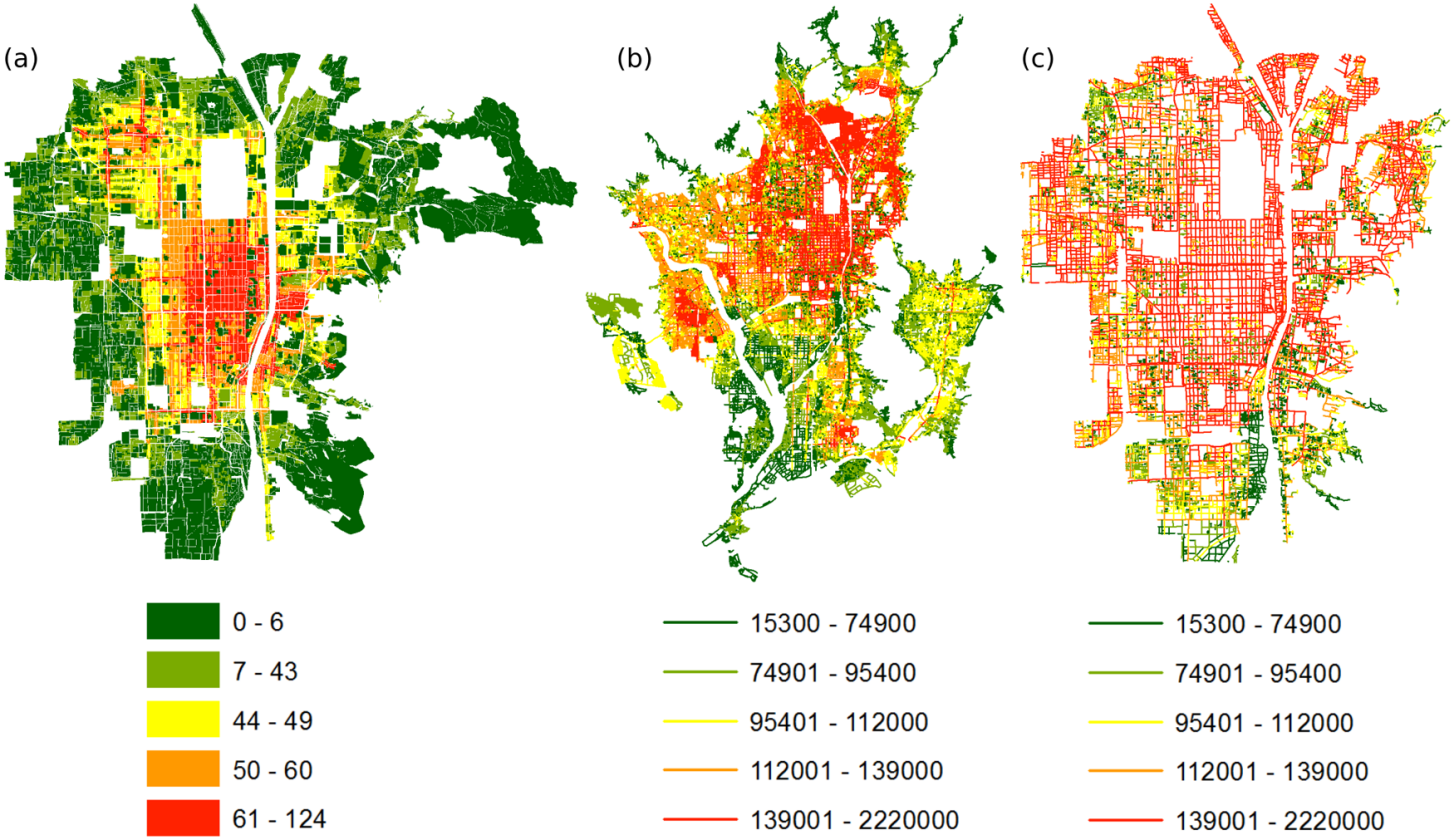
Study areas. (a) Kyoto, 1912 extent; (b) Kyoto, 2012 extent; (c) 2012 Kyoto cropped to the 1912 extent. All prices are expressed in Yen (1 Yen of 1912 is worth approximately 2000 Yens of 2012).

For present Kyoto, the data available is presented as land tax assessed value by road valuation around 2012, as found on the Kyoto Open Data website provided by Kyoto City. The price is given as mean land price per square meter along each road segment, where a road segment is defined as any part of a road included between two street intersections (or dead ends). Most road segments are edges of a block, and some are smaller intra-block streets. Making the unavoidable assumption that the difference in depth between different residential buildings is negligible compared to the length of the roads segment, we have multiplied the mean price along each segment by the length of said segment. The result are values proportionate to the sum of all land lot prices along each segment. There is a total of 41524 values. Since multifractals are invariant under a linear transformation, those values can be seen as approximations comparable to 1912 lot values (although at a broader scale). Both the full extent of modern Kyoto (S2 Dataset) and its intersection with the extent of 1912 Kyoto (12000 values, S3 Dataset) will be considered. All study areas are represented in Fig 3.

For both datasets, values have been paired with the centroids of their corresponding lots or road segments. The data is processed as a matrix representing a grid overlay over the Kyoto map. Due to the relatively broad scale (especially for present Kyoto), to the interference created by simplification during the price assessment process, and to the messy nature of real estate, we do not expect that the data will be a clean multifractal with definite self-similarity at all scales. For that reason we use the multiplier method to calculate in a sense the “best fit” spectrum. Even if the data is not exactly self-similar at all scales, the multifractal methodology allows nonetheless a consistent comparison of the variety and spatial partitioning of prices between different city models as long as the data shares a similar resolution and structure. The results for present Kyoto are to be taken with more caution than those for Taisho era Kyoto because of the difference in data quality. Similarly, as evidenced in the previous section, the part of the spectra for *α* values below 2 is more reliable and expected to be in better accordance with more “rigid” multifractal methods than the part for *α* values greater than 2.

## Results

Urban models were used to analyse the influence of changing the spatial distribution or the price distribution on the resulting multifractal spectra. We first present a detailed analysis for Taisho era Kyoto and its associated models, then a more concise analysis for present Kyoto due to the less appealing shape of the data. We finally make a temporal comparison between both datasets, and use the PLUTO database for New York and Land Registry house transactions database for London to compare the results with these two cities. Technical aspects of the urban models creation and isolated spectra for each type of model are provided in more detail in Appendix A1 in S1 Appendix. While we focus here on the multifractal spectra alone, the so-called generalized dimension usually associated to multifractal analysis is indicated in Appendix A2 in S1 Appendix.

### City models: 1912 data

**Fig 4:**
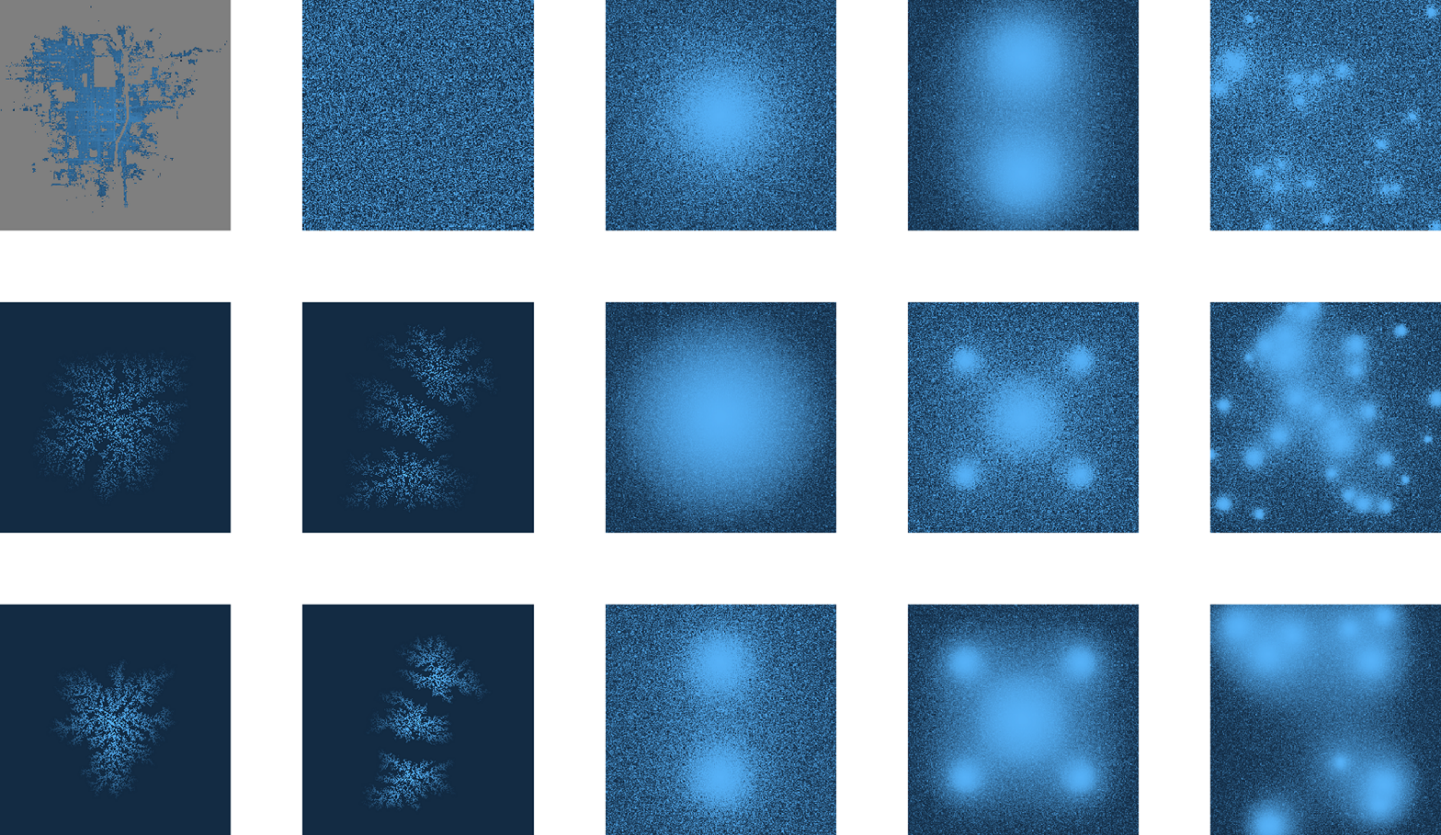
Spatial models. Top left: logarithmic overlay of the real price distribution over the real Kyoto pattern in a grid of resolution 256x256. Next to it: corresponding rank distribution drawn uniformly. Bottom left: rank distribution overlayed over four DLA models with 1 or 3 centres and different levels of attraction. Right: rank distribution overlayed over nine polycentric models with different number of centres and levels of attraction.

A first batch of models is built by changing the spatial pattern while the price distribution is kept identical to the true distribution. Three types of spatial distributions are chosen to supplement the true pattern: uniform, polycentric, and diffusion-limited aggregation (DLA). The first one is used as a null model, the second one as a representation of how modern megacities develop [23–25], and the third one as a multifractal reference since it is known to generate strong multifractality [26–29]. All these models are plotted in Fig 4 alongside the true distribution. The latter is represented in the top left image. Next to it is the price distribution drawn uniformly. The four figures on the bottom left are DLA models with either 1 or 3 centres exerting different levels of attraction. Finally, the nine models on the right are polycentric models with different number of centres exerting varying levels of attraction. The true spatial distribution uses a logarithmic overlay of the real price distribution, while all other images use an overlay representing the rank of each point in the price distribution after it has been drawn into the space. All images are represented in a grid of resolution 256x256.

The uniform distribution has been drawn 50 times. To generate the polycentric models, a number of centre coordinates (between 1 and 30) are chosen randomly in the grid. Then, all the cells in the grid are ranked according to the formula

$$\begin{array}{c} s_{i}=\sum_{k}b_{k}/d_{ik}^{\gamma }, \end{array}$$ (6)

where *s*_*i*_ represents the strength of cell *i*, *b*_*k*_ a weight given to centre *k*, *d*_*ik*_ the distance between point *i* and centre *k*, and *γ* a global “attractivity” parameter. The number of centres and value of *γ* for each case are given in the appendix. Similarly, the DLA models are generated by first choosing randomly either one or three “anchor” coordinates in the grid. Then, the cells are ranked by introducing “particles” carrying a rank tag (lowest ranks first) into the system. Each particle operates a random walk until it encounters an anchor or a cluster of particles attached to an anchor, and attaches to it with a probability of either 1 or 0.5. The results of the DLA process are available in S4, S5, S6 and S7 Datasets.

Once all the cells in the grid have been ranked (ties are resolved by a random draw), the prices are mapped over the points by rank order, so that the most expensive prices are closer to the centres or anchors. A noise is previously introduced in the ranked price distribution, by redrawing the price ranks according to a probability distribution

$$\begin{array}{c} r_{i}^{p}/\sum r_{i}^{p}, \end{array}$$ (7)

where *r*_*i*_ is the current rank in the distribution, and *p* is a power set to 2 for the polycentric models, and either 1 or 8 for the DLA models. See the appendix for additional parameter choices compared to the selection presented here.

A second batch of models is built by changing the price distribution while the spatial pattern is kept identical. Three distributions are considered: uniform, truncated normal, and Pareto. The uniform distribution is taken as a null model, while the normal and Pareto distributions are the most recurrent distributions observed in urban science (in particular, the Pareto distribution is usually associated to the distribution of wealth). The true distribution is log-normal. To create the distributions, range, mean and standard deviation can be adjusted. Since it is by construction impossible to match all three parameters for each distribution, priority was given to matching the range with the true distribution. When the exceptionally high priced imperial palace is removed, this range consists of values between 0 and 10000 yens. These three distributions are plotted in Appendix A1 in S1 Appendix. In addition to the price distributions being laid over the true spatial pattern, they have also been laid over the uniform spatial distribution for reference.

**Fig 5:**
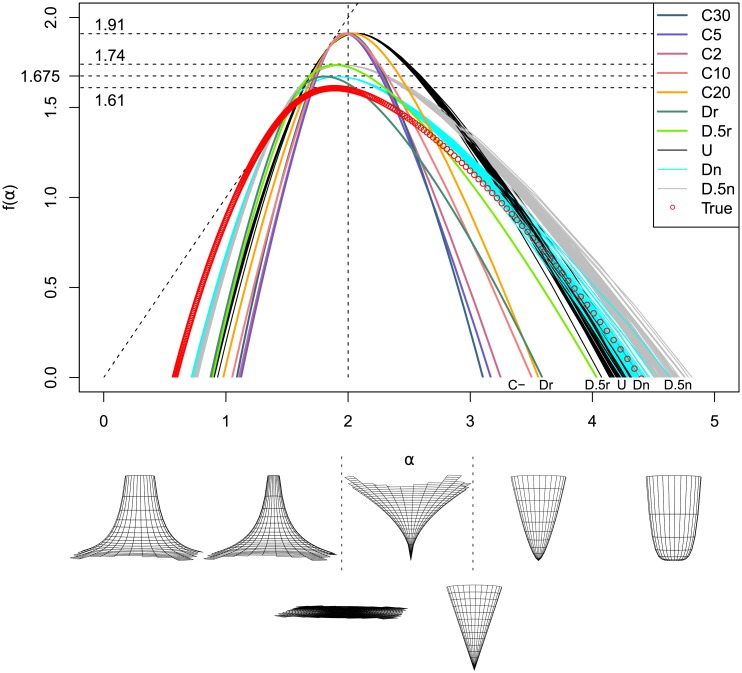
Spectra for 1912 Kyoto price distribution mapped over several spatial models. The models are polycentric (C-, where—is the number of centres), ranked DLA (Dr), ranked DLA with half centre attraction (D.5r), uniform (U, 50 draws), DLA with a noise (Dn, 50 draws), DLA with half centre attraction and a noise (D.5n, 50 draws), and true distribution (True). The corresponding idealized two-dimensional signal is added below the *α* axis for reference.

The spectra resulting from the first batch of models plotted against the true Kyoto distribution can be seen in the top of Fig 5. Only the most relevant cases were selected, and some additional curves for each type of model can be found in Appendix A1 in S1 Appendix. We have in total: six polycentric models (referred to as *C*− in the legend, where − is the number of centres), fifty iterations of the uniform draw (*U* in the legend), and two fully ranked one-centre DLA models with normal and halved centre attraction weights and fifty iterations of each DLA model with a noise added to it (respectively *Dr*, *D*.5*r*, *Dn*, and *D*.5*n*). We can immediately observe that the real distribution (red circles) seems to maximize the width of the spectrum, while minimizing its height. Only some of the DLA models with added noise could create a wider right-spectrum and remain close on the left side. Polycentric models generate fairly weak multifractality, especially considering that the multiplier method tends to artificially widen the spectrum in weak cases. The uniform distribution lays between the polycentric and DLA distributions.

**Fig 6:**
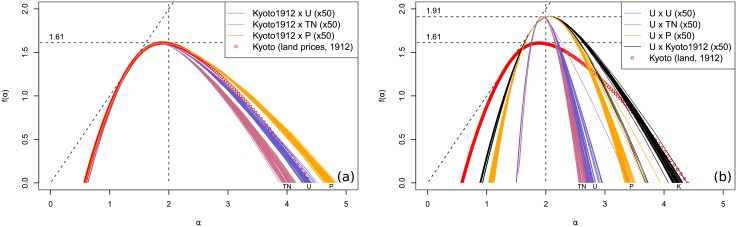
Spectra for 1912 Kyoto and price models. (a) Uniform (U), truncated normal (TN), Pareto (P) and true (red circles) distributions mapped over 1912 Kyoto true spatial distribution. (b) Same distributions and true price distribution (K) uniformly drawn into space.

Since the price distribution is the same for all models, we can conclude that the spatial distribution has a large impact on shaping the width of the spectrum. This is further corroborated by the second batch of models for which several price distributions are mapped over the true distribution and over the uniform distribution (Fig 6). In the first case, there is almost no difference in the left part of the spectra, while the right part are very similar, with Pareto distribution giving the widest spectrum. However, the price distribution does have an impact on the spectrum, as evidenced by the second case, where all four price distributions have been drawn randomly in space. The uniform and truncated normal distributions present almost no multifractality, the very narrow spectrum being identifiable to an artefact of the multiplier method. The Pareto and the real distributions produce more convincing spectra, close to one another.

### City models: 2012 data

**Fig 7:**
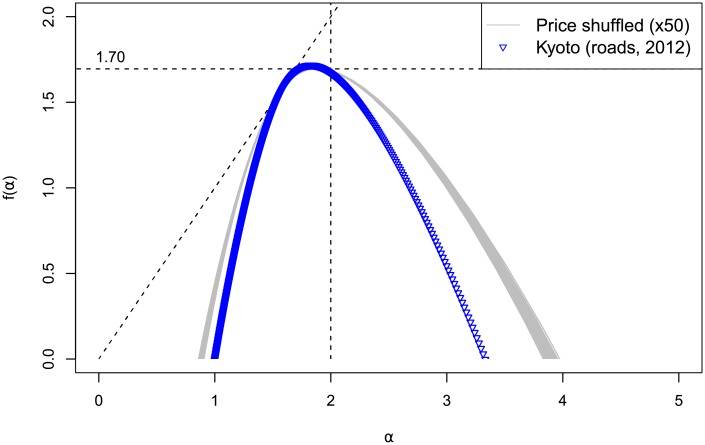
Spectra for 2012 Kyoto and shuffling. Spectra for 2012 Kyoto price distribution shuffled inside the true spatial pattern (50 draws) compared to the true 2012 distribution (blue triangles).

Recall that for present Kyoto, the processed data consists of centroid coordinates for each road segment in Kyoto. To each centroid is attributed the mean square meter price multiplied by the length of each corresponding segment. Instead of constructing many less reliable and redundant models compared to the ones built for Taisho era Kyoto, we focus on shuffling randomly the price distribution in a space where all the actual locations of road segment centroids are preserved. Fifty iterations of this process were done and produced the spectra that can be seen in the top of Fig 7. Contrary to the 1912 case, the real placement seems to minimize the spectrum width.

**Fig 8:**
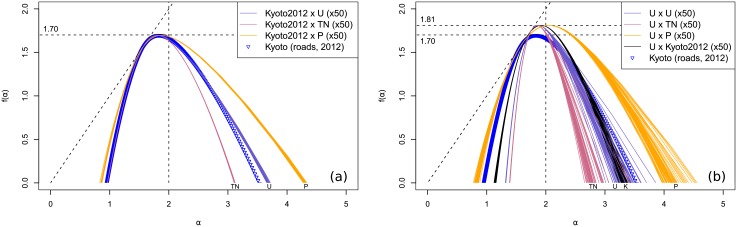
Spectra for 2012 Kyoto and price models. (a) Uniform (U), truncated normal (TN), Pareto (P) and true (blue triangles) distributions mapped over Kyoto true spatial distribution in 2012. (b) Same distributions and true price distribution (K) uniformly drawn into space.

The price distribution for 2012 Kyoto is almost log-normal, akin to the price distribution for 1912, although it is less symmetrical and only spans over two orders of magnitude compared to four in the previous case. Similarly, we generated 50 iterations of some corresponding uniform, normal and Pareto distributions. The spectra for each distribution can be found mapped over the true spatial pattern in Fig 8a, and uniformly drawn into space in Fig 8b. The results are similar to those obtained for 1912. In the true pattern case on the left, the spectra follow the same ranking in width as for 1912, with only slightly more pronounced differences. In the uniform pattern case on the right, the actual price distribution gives results closer to the uniform distribution, while the Pareto distribution provides significantly wider results. This is coherent with the narrower range and higher maximum of the true price distribution, reflected in a more dispersed corresponding Pareto distribution.

### Comparing Kyoto across time, and with data from other cities

To be able to compare accurately Kyoto in 1912 and in 2012, it would have been preferable to use the same resolution in both cases. Unfortunately, the road valuation data for 2012 creates undesired “gaps” between data points when plotted at the resolution used in the 1912 case. Those gaps do not describe real land value well, as price lots should be contiguous or almost contiguous, with only big natural obstacles (such as the Kamo river for Kyoto) representing an actual empty space. To tackle this problem, the resolution used for present Kyoto has been reduced by 4 to obtain a relatively compact grid. A first map was created representing the full extent of the present city at a resolution of 512x512, the same resolution that was used for the 1912 extent of the city, despite the new city being roughly four times bigger than the old city. A second map was created to represent the evolution of the old city alone. It comprises only the roads that are fully included in the boundaries of the 1912 city. This map is at a resolution of 256x256, i.e. four times lower than for the 1912 map, despite the geographical extent being identical. The study areas for each case are illustrated in Fig 3.

**Fig 9:**
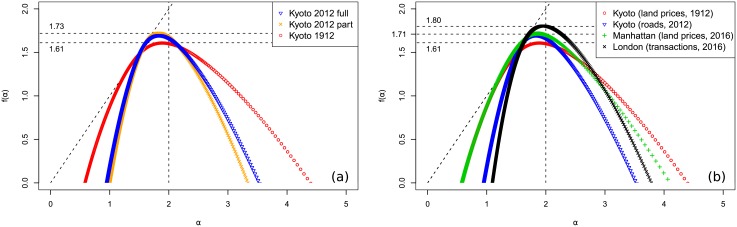
Kyoto through time and compared to London and New York. (a) Comparison between Kyoto price distribution in 1912 and in 2012 (blue triangles for the full extent, orange crosses for the 1912 boundaries). (b) Comparison between Kyoto land price distribution in 1912, Kyoto road valuation in 2012, Manhattan land price distribution in 2016, and London house transaction prices in 2016.

The spectra for both maps are plotted against the 1912 spectrum in Fig 9a. It could be hypothesized that the increase in spectrum height for the modern city is representative of densification. Indeed, the eastern half of the city is much more compact in 2012 than it was in 1912. Unfortunately, it can also be an artefact of the lower resolution, so no definite conclusions can be drawn.

The evolution of the width of the spectrum is more interesting. Since summing measures defined on disjoint supports will result in a spectrum for which *f*(*α*) is the maximum of the *f*(*α*) in the spectrum of each independent measure, it was expected that the spectrum corresponding to the 1912 boundaries map would be narrower than the spectrum for the full present city. Adding newly developed zones to the old centre can only add variety and make the spectrum wider. The fact that it is only slightly narrower indicates that there is no significant discrepancy in the development of new neighbourhoods compared to how the old city evolved.

Comparing the 2012 spectrum to the 1912 spectrum, there is an observable loss of extreme *α* values, corresponding to the steepest spatial increase in price, while the relatively homogeneous zones, i.e. those around *α* = 2, are of higher *f*(*α*) dimensions in the 2012 case. This is representative of a noticeable increase in local homogeneity. It can also be noted that the shape is more symmetric in 1912 compared to 2012, with a rounder left half of the spectrum. It means that the diversity in 2012 is created more by (small) local “bumps”, and less by (small) local “gaps” compared to 1912.

The 2016 PLUTO database for New York contains the assessed tax value for each of the 43k lots in Manhattan for the 2017 fiscal year. According to the documentation, it is “calculate[d] by multiplying the tax lot’s estimated full market land value, determined as if vacant and unimproved, by a uniform percentage for the property’s tax class” by the Department of Finance. It is quite similar to the assessed land lot value from the Kyoto 1912 dataset. The resulting spectrum can be found in Fig 9b. It can be observed that it is surprising close in width to the spectrum for 1912 Kyoto, particularly on the right side. It is also similar in height to 2012 Kyoto. A look at the values from year to year in the PLUTO database (which starts in 2002), indicates that the prices are quite stable through time, with only small readjustments from one year to another. It can be hypothesize that the 2017 assessed land prices distribution is in reality close to the early 20th century’s distribution, explaining the similarity with 20th century Kyoto. On the other hand, the *D*_0_ value representing the fractal dimension of the physical city, is closer to present Kyoto.

Another dataset containing all the house transactions that happened in London in 2016 can be obtained from the Land Registry. For that year, there were a little under 100k transactions. Although house transactions are not directly comparable to land prices, it gives an idea of the real estate market in London for that particular year. The resulting spectrum is plotted in Fig 9. While Manhattan data produced a spectrum close to Taisho era Kyoto, the data for London in 2016 produces a result that is convincingly close to present Kyoto, particularly for the left (most stable) half of the spectrum. This trend of multifractal loss in modern cities has already been observed for road networks [11, 12, 30].

Overall, the wide spectrum obtained for the real cases and the ability to discriminate between situations, indicate that the multifractal methodology can provide informative insights when applied to land prices measures. It can be interesting to relate the results with the information (or entropy) dimension which corresponds to the point of the curve for which *α* = *f*(*α*). The information dimension can be related to Shannon’s entropy and provides information on the density evenness (or sparseness) in the data, with smaller values corresponding to more heterogeneities. As can be seen from the curves, where it corresponds to the intersection with the dashed identity line, this dimension is consistent with the spread of the curves. The entropy dimension is part of the generalized dimension, and has also been calculated by a direct approach in appendix A2 in S1 Appendix. All the values calculated in that way are consistent (in particular the striking similarities between Kyoto, London and New York), except for the uniform model that unexpectedly has the smallest value. It must finally be noted that the results are in accordance with Hu et al. [5] who also found multifractality for land price distributions in Wuhan City in China. We will show in the next section that the results agree with classical inequality indicators, legitimating its use in an urban inequality context.

## Discussion

We compare the results obtained from multifractal analysis with the results obtained when the classical inequality and segregation indicators are applied to the data. We also point out the necessity to contextualize the analysis with inter-measure comparison instead of inter-city comparison alone.

### Comparison with classical indicators

In their review of existing economic segregation measures, Reardon et al. [1] (see also [31]) distinguish three different groups of measures. The first group, category-based measures, does not take into account the ordinal nature of the variable, and is irrelevant to our study (see [32]) for an extensive compilation of such indices). The second group, variation-ratio measures, such as the Neighbourhood Sorting Index (NSI) [33, 34], compares the ratio of the between-neighbourhood variation compared to the total population variation for some definitions of variation [35–37]. The third group, spatial measures, such as an adapted version of geographical autocorrelation [38, 39], takes into account the spatial patterning of the variables. It is the most interesting and least well developed one according to them.

The multifractal methodology would pertain to this last group and would not be concerned with the flaws underlined by Reardon et al for the other measures. Indeed, it is not subject to the arbitrary nature of the definition of geographical unit or categorical thresholds, and it is insensitive to linear transformations (and moderately sensitive to affine transformations using the multiplier method) in the variable distribution. To avoid these flaws, Reardon et al. had developed new spatial measures based on the variation-ratio idea, among which are the Ordinal Information Theory Index (OITI) and Ordinal Variation Ratio Index (OVRI). We will try to demonstrate the advantages of the multifractal approach over these measures.

In addition to the segregation NSI, OITI and OVRI indices, we consider three a-spatial inequality measures: the relative dispersion (RD), the popular Gini coefficient, and the Theil index [40]. The formal definitions for all six indices can be found in the supporting information Appendix A3 in S1 Appendix. For the practical computation, Kyoto is arbitrarily divided in 100 square neighbourhoods.

The results for 1912 Kyoto are shown in Table 1 for the full residential land price distribution (Tot.), for a partial price distribution (Part.) that excludes a few outstandingly expensive lots (such as the Imperial palace), as well as for the uniform (Unif.), truncated normal (TN) and Pareto (Pareto) price distributions mapped over the true spatial distribution. In accordance with the multifractal analysis, the RD, Gini, Theil and NSI coefficients provide similar values between the partial and Pareto distributions on the one side, and between the truncated normal and uniform distribution on the other side. Also in accordance with the multifractal analysis, the RD and Gini coefficients indicate more distributional variety for the partial and Pareto distributions than for the uniform and truncated normal ones. According to a remark in the methodology part, this translates into higher levels of spatial segregation for the truncated normal and uniform distributions, which is indicated both by the spectra of Fig 6 and the values of the NSI coefficient. It is noteworthy that the NSI coefficient is made irrelevant for the full price distribution. Indeed, its definition offers no counter to the Imperial palace making all other lots negligible compared to it. In contrast, multifractal analysis is more resilient to unique outstanding value, which can only add to the total variety. Finally, the OITI and OVRI indicate no difference between all price models. This insensitivity is by construction. We prefer the multifractal analysis ability to pick up differences between price distributions even if the spatial pattern is the dominant element determining the shape of the spectrum.

**Table 1:** Classical inequality measures for different price distributions (Kyoto, 1912).

Indicator	Tot.	Part.	Price: Unif.	Price: TN	Price: Pareto
RD	0.998	0.904	0.498	0.402	0.905
Gini	0.666	0.606	0.332	0.281	0.610
Theil	1.833	0.786	0.192	0.133	0.734
NSI	0.033	0.234	0.500	0.502	0.338
OITI	0.147	0.147	0.147	0.147	0.147
OVRI	0.166	0.165	0.166	0.166	0.166

Another set of results is presented in Table 2. The partial 1912 distribution has been mapped over the uniform spatial distribution (Unif.), over one centre and five centres polycentric models (C1A4 and C5A1B3), and over one seed and three seeds DLA models (DLA1 and DLA3). None of the indicators show segregation for the uniform distribution, which is expected. OITI and OVRI failed to distinguish between C1A4, DLA1 and DLA3 models, and NSI between C1A4 and DLA1 models. The C5A1B3 model shows significantly less segregation than the C1A4 model because it is made roughly of 5 copies of the same concentric distribution, even though the repartition would feel identical from an inhabitant perspective. This shows that these measures are not completely free from the modifiable areal unit problem (MAUP), contrary to the multifractal analysis.

**Table 2:** Classical inequality measures for different space distributions (Kyoto, 1912).

Indicator	Part.	Space: Unif.	C1A4	C5A1B3	DLA1	DLA3
NSI	0.234	0.043	0.530	0.396	0.451	0.241
OITI	0.147	0.004	0.480	0.170	0.488	0.451
OVRI	0.165	0.002	0.442	0.155	0.511	0.476

The results for 2012 Kyoto are shown in Table 3 for the entire dataset (Tot.), for the part of the dataset that corresponds to the extent of the 1912 city (Part.), for a shuffling of the full dataset (Shuffled), and for the uniform (Price: Unif.), truncated normal (Price: TN), and Pareto (Price: Par.) distributions mapped over the real city. As found previously and as expected, the OITI and OVRI coefficients are invariant through change of price distribution, while the RD, Gini and Theil coefficients are invariant through shuffling. Overall, the same decrease of inhomogeneity between 1912 and 2012 is picked up by all coefficients, especially the spatial ones (NSI, OITI, OVRI). Contrary to the multifractal analysis, the shuffled distribution appears more homogeneous than the real distribution, which is true at the broader scale used to compute the NSI, OITI and OVRI coefficients, but not at the microscale used for multifractal analysis. Also contrary to the multifractal analysis, the partial distribution is slightly more unequal than the total one. This is due to the property of multifractal analysis that the spectrum of the total measure must be wider than the spectrum of a part of it. In a sense, multifractality “adds” all the variety in the measure whereas the classical indicators are “rescaled” inside each subset. Finally, the ranking between price distributions is similar to the one found in Fig 8.

**Table 3:** Classical inequality measures for different space distributions (Kyoto, 2012).

Indicator	Tot.	Part.	Shuffled	Price: Unif.	Price: TN	Price: Par.
RD	0.701	0.768	0.701	0.498	0.282	0.991
Gini	0.479	0.520	0.479	0.332	0.199	0.661
Theil	0.400	0.479	0.400	0.192	0.067	1.02
NSI	0.267	0.376	0.044	0.251	0.260	0.203
OITI	0.041	0.074	0.002	0.041	0.041	0.041
OVRI	0.043	0.079	0.002	0.043	0.043	0.043

Overall, almost all conclusions that could be drawn from the classical indicators are in accordance and could also be found with the multifractal analysis. The latter also allowed us to obtain more information and was found to be better at distinguishing some of the spatial models (in particular the polycentric one) and at giving a result more coherent with the intuitive perception of those places.

### The need to relate results to other measures

Contrary to intuition, a narrower spectrum, which means less variety, is not necessarily equivalent to less inequality. As a matter of fact, a narrow spectrum with a wide price range means important segregation, since it indicates that the many price bands are all grouped in locally relatively homogeneous environments. Furthermore, it could appear better suited that the land price distribution matches the income distribution to ensure maximum affordability instead of a situation where all prices are equally unaffordable. Unfortunately, income data tends to be scarcer and usually comes divided into broad categories that are unsuitable for multifractal analysis. Nonetheless, the multifractal results are in accordance with recent research suggesting that segregation in modern Japanese cities is expected to be particularly low. Some social explanations (that are beyond the scope of this article) have been proposed by Fujita and Hill [41], an article that was thoroughly reviewed by Fielding [42].

It would also be interesting to relate accessibility to land prices. Whether a wider or narrower spectrum should be aimed for accessibility would depend on the variable considered: a node network with a wider spectrum offers more option to travel from one point to another with effective hubs, while a time length network should be as narrow and centred over *α* = 2 as possible to avoid exponentially long journeys to particular places.

## Supporting information

S1 Appendix Technical precisions and additional descriptors. Contains details on the technical implementation of the analysis, tables showing the generalized dimension for the city models, and the formal definitions of the inequality indicators.
(PDF)

S1 Dataset 1912 Kyoto land prices. (CSV)

S2 Dataset 2012 Kyoto land prices. (CSV)

S3 Dataset 2012 Kyoto cropped to the 1912 extent land prices. (CSV)

S4 Dataset DLA model with 1 centre. (CSV)

S5 Dataset DLA model with 1 centre and a sticky probability of 0.5. (CSV)

S6 Dataset DLA model with 3 centres. (CSV)

S7 Dataset DLA model with 3 centres and a sticky probability of 0.5. (CSV)

S1 Codes Txt transcript of the R codes used for the analysis. (TXT)
