# Supplementary material for: Uncovering inequality through multifractality of land prices: 1912 and contemporary Kyoto
Source: PLoS One. 2018 Apr 30;13(4):e0196737. doi: 10.1371/journal.pone.0196737 (PMC5927455; doi:10.1371/journal.pone.0196737)
Supplement: S1 Appendix — Contains details on the technical implementation of the analysis, tables showing the generalized dimension for the city models, and the formal definitions of the inequality indicators. (PDF) [file pone.0196737.s001.pdf]

Uncovering inequality through multifractality of land prices: 1912 and contemporary Kyoto - Appendices

Hadrien Salat[1*], Roberto Murcio[2], Keiji Yano[3], Elsa Arcaute[1]

1 Centre for Advanced Spatial Analysis, University College London, London, United Kingdom
2 Consumer Research Data Centre, University College London, London, United Kingdom
3 Department of Geography, Ritsumeikan University, Kyoto, Japan

* hadrien.salat@ucl.ac.uk (HS)

Appendix A1: Technical aspects of the multifractal calculations and models creation

In our context, boxes are defined as Moore neighborhoods of radius r around each point such that a full box can be included inside the study area. In practice, this means that points too close to the border to fit a box of maximal size were ignored in the calculations. The values $\mu_i(r)$ are then the sum of all the lot price whose centroid falls inside each box i. The chosen radius r_0 was the minimum allowed by the resolution (i.e. 1 pixel), and we averaged the results over several r_k to limit the effect of local inaccuracies in the data. The q range is defined as all values such that the resulting $f(\alpha)$ is non-negative.

Recall

$$\tau(q) := \alpha(q)q - f(\alpha(q)) \approx \lim_{r\to 0} \frac{\log(Z(q,r))}{\log(r)}. \tag{1}$$

and

$$\tau(q) + d \approx -\frac{\log\left(1/N \sum_i M_i^q\right)}{\log(r_k/r_0)}; \tag{2}$$

$$\alpha(q) \approx -\frac{\sum_i M_i^q \log(M_i)}{\sum_i M_i^q \log(r_k/r_0)}. \tag{3}$$

As defined above in equation 1, the $\tau(q)$ values should yield $\tau(1) = 0$ and $\tau(0) = -d_0$, where d_0 is the box-counting dimension of the support of the measure μ, so that the spectrum touches the identity line for $q = 1$, and so that its maximum is d_0, achieved for $q = 0$. However, by construction, the right-hand part of equation 2 returns 0 for $q = 0$, and a value close to d for $q = 1$, where d is the dimension of the space A. The expected τ values are somewhat restored by the addition of d in the left-hand part of equation 2. Since we find more

meaningful to obtain d_0 instead of d for the spectrum maximum, we have added a rescaling by d_0/d to equations 2 and 3 in order to recover the usual $\tau(1) = 0$ and $\tau(0) = -d_0$.

The uniform distribution (Fig 1) has been drawn 50 times. The nine polycentric models (Fig 2) are created by ranking each cell in a grid following equation

$$s_i = \sum_k b_k / d_{ik}^{\gamma}, \tag{4}$$

where s_i represents the strength of cell i, b_k a weight given to centre k, d_{ik} the distance between point i and centre k, and γ a global "attractivity" parameter. They are labelled in the legend starting with the letter C followed by the number of centres. The number following the letter A indicates the gravitational power γ, and the letter B indicates that the centres were given different (random) barycentric weights. A noise

$$r_i^p / \sum r_i^p, \tag{5}$$

with $p = 2$ here, has been added to the price rankings.

The DLA distributions (Fig 3, 4, 5 & 6) have first been drawn once in a configuration where the particles representing land lots are liberated into the system according to their rank in the price distribution (most expensive lots being sent first). Then two types of noise, corresponding to equation 5 with $p = 1$ and $p = 8$, were added (50 times each) to the price rankings. In the legend, $DLAx(/y)$ refers to a DLA model using x seeds and a sticky coefficient of y, Noise$x$ refers to the noise power.

The true, uniform, normal and Pareto price distributions used for comparison in the 1912 case are represented in Fig 7, and those same distributions for the 2012 case are represented in Fig 8.

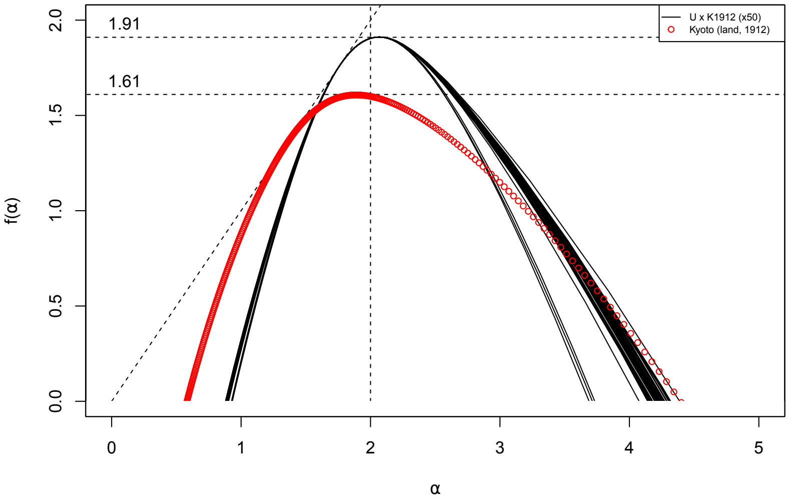

Figure 1: Multifractal spectrum for Kyoto 1912 price distribution drawn uniformly 50 times.

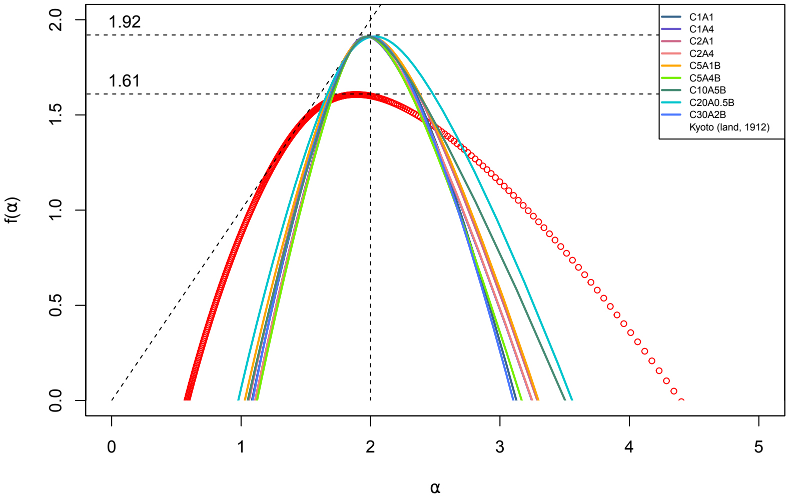

Figure 2: Multifractal spectrum for Kyoto 1912 price distribution mapped on polycentric space distributions. The number after C is the number of centres, the number after A is the global attractivity of centres, and B indicates that centres have different attraction weights.

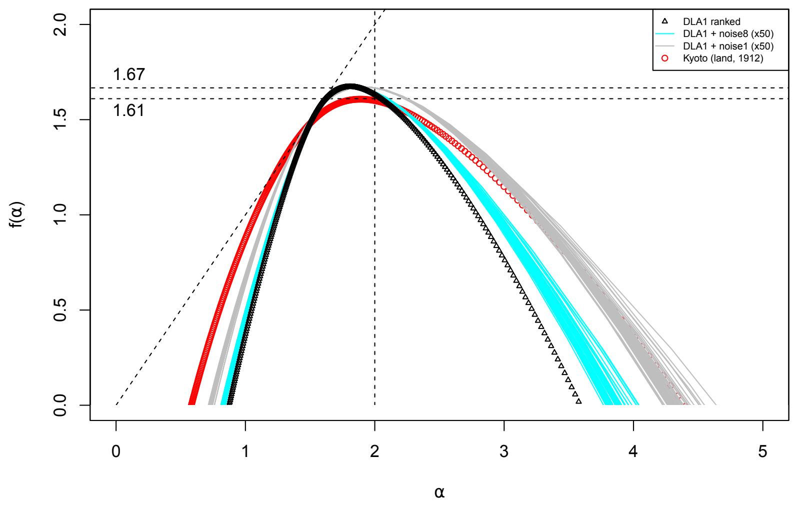

Figure 3: Multifractal spectrum for Kyoto 1912 price distribution mapped on a DLA with 1 centre.

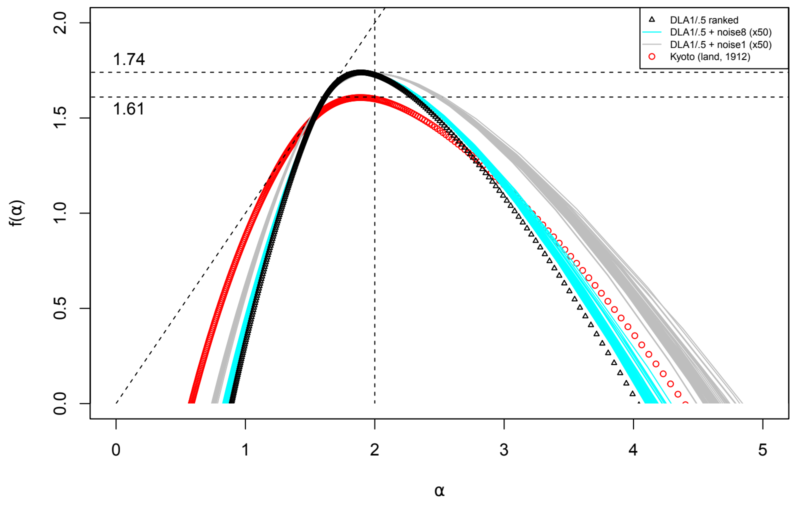

Figure 4: Multifractal spectrum for Kyoto 1912 price distribution mapped on a DLA with 1 centre and sticky probability of 0.5.

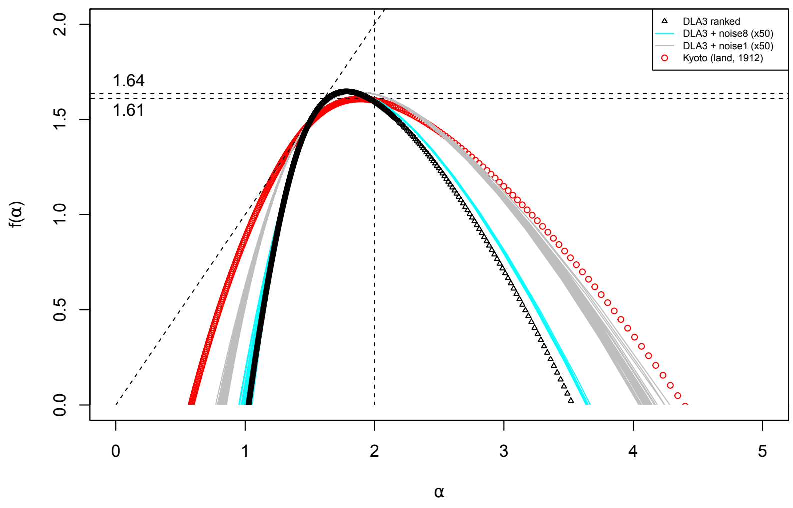

Figure 5: Multifractal spectrum for Kyoto 1912 price distribution mapped on a DLA with 3 centres.

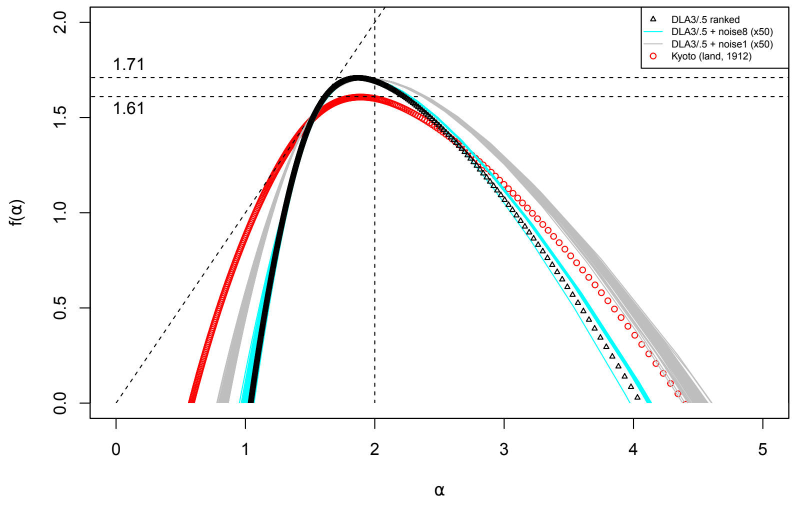

Figure 6: Multifractal spectrum for Kyoto 1912 price distribution mapped on a DLA with 3 centres and sticky probability of 0.5.

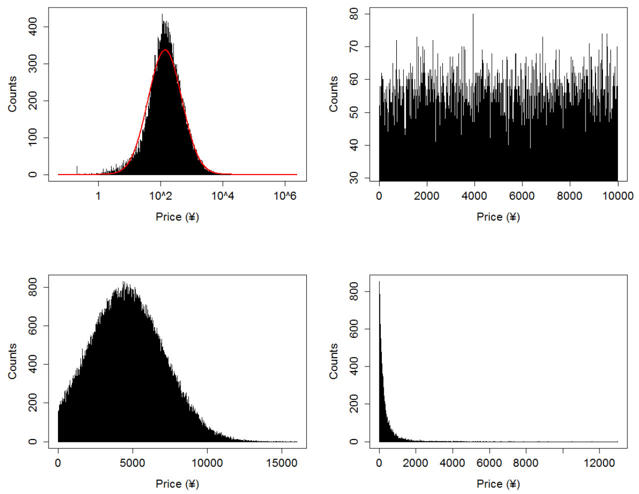

Figure 7: 1912 price distributions. Top left: real Kyoto price distribution (note the logarithmic x-axis), top right: uniform distribution, bottom left: truncated normal distribution, bottom right: Pareto distribution.

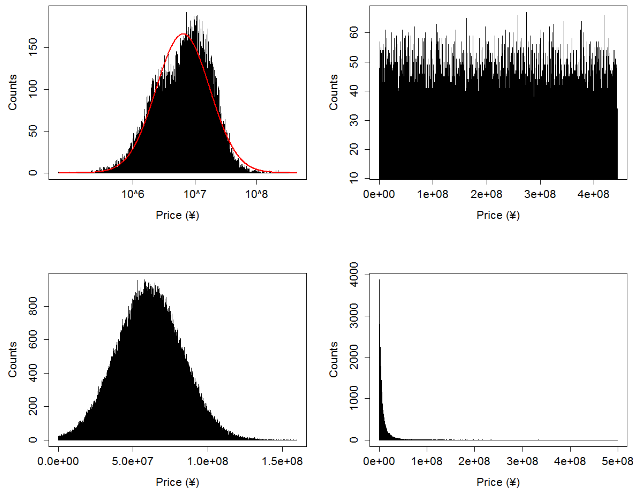

Figure 8: 2012 price distributions: real (note the logarithmic x-axis) and corresponding uniform, truncated normal, and Pareto price distributions.

Appendix A2: Tables of D0, D1 and D2 for all cases

Among the generalized dimension defined by the equation $D(q) := \tau(q)/(q-1)$, three values relate to well known dimensions: $D(0)$, $D(1)$ and $D(2)$. The first one, $D(0)$, is the fractal dimension of the physical space supporting the measure. The dimension $D(1)$ is the information (or entropy) dimension, it relates to Shannon's entropy and provides a measure of the density evenness in the data. Finally, $D(2)$ is the correlation dimension, which provides a measure of scattering in the data. By definition, the full range of $D(q)$ is the same as the full range of α. The $D(0)$ dimension can be computed directly using box-counting, while the $D(1)$ and $D(2)$ values can be deduced from the direct expressions

$$D(1) = \lim_{r \to 0} \frac{\sum_i \mu_i(r) \log(\mu_i(r))}{\log(r)}, \tag{6}$$

$$D(2) = \lim_{r \to 0} \frac{\log(\sum_i \mu_i(r)^2)}{\log(r)}. \tag{7}$$

The D_0, D_1 and D_2 values for Kyoto in 1912, 2012 and 2012 intersected with 1912 boundaries, for the uniform, polycentric and DLA models for 1912 data and shuffled model for 2012 data, as well as for Manhattan assessed tax land value in 2016 and London house price transactions in 2016 can be found in table 1. Those values show the same decrease in multifractality for modern data as the spectra in the main text. Three problematic values with slightly higher D_1 than D_0 have been obtained for Kyoto in 2012 (1.74 vs 1.70), DLA 1 ranked (1.70 vs 1.67) and DLA 1.5 ranked (1.78 vs 1.74).

Table 1: **D_0, D_1 and D_2 values for the main city models. (Kyoto, 1912).**

Model	D_0	D_1	D_2
Kyoto 1912	1.61	1.50	0.23
Kyoto 2012	1.70	1.74	1.70
Kyoto 2012 (part.)	1.72	1.70	1.58
Uniform (1912)	1.91	1.34	0.03
Polycentric (1912)	1.91	1.57	0.14
DLA 1 (ranked)	1.67	1.70	1.23
DLA 1.5 (ranked)	1.74	1.78	1.35
DLA 1 (noise)	1.67	1.62	0.98
DLA 1.5 (noise)	1.74	1.54	0.70
Shuffled (2012)	1.68	1.51	1.37
Manhattan 2016	1.71	1.51	1.37
London 2016	1.80	1.76	1.60

Appendix A3: Formal definitions of the classical inequality indicators used

Denote $\{x_i\}_{1\leq i\leq n}$ the set formed by n observations, μ their average value and p_i the associated probability distribution defined as $p_i = x_i/\sum_j x_j$. Then, the relative dispersion (RD) is defined as

$$\mathrm{RD} = \frac{\sum_i |\mu - x_i|}{n\mu}. \quad (8)$$

The Gini coefficient (G) is defined as

$$\mathrm{G} = \frac{1}{2}\frac{n^2\mu}{\sum_i \sum_j |x_i - x_j|}. \quad (9)$$

The Theil coefficient (T) is defined as

$$\mathrm{T} = \sum_i p_i \log(np_i). \quad (10)$$

Now, assume the space is divided into n neighbourhoods, denote $\{n_k\}_{1\leq k\leq n}$ the population count inside each neighbourhood, and $\{\mu_k\}_{1\leq k\leq n}$ the average value inside each neighbourhood. Then, the neighbourhood Sorting Index (NSI) is defined as

$$\mathrm{NSI} = \sqrt{\frac{\frac{\sum_k n_k(\mu_k-\mu)^2}{n}}{\frac{\sum_i (x_i-\mu)^2}{n}}} \quad (11)$$

For the indices defined by Reardon et al., it is needed to define first a segregation measure S comparing inter-neighbourhood variation and total variation by

$$S(v) = \sum_{k=1}^{N} \frac{n_k}{nv}(v - v_k), \quad (12)$$

where v is a chosen variation function, and v_k is its value inside neighbourhood k. The Ordinal Information Theory Index (OITI) and Ordinal Variation Ratio Index (OVRI) are defined respectively for the following variation functions v_1 and v_2

$$v_1 = \frac{1}{K}\sum_{i=1}^{K} -[c_i \log(c_i) + (1 - c_i)\log(1 - c_i)]; \quad (13)$$

$$v_2 = \frac{1}{K}\sum_{i=1}^{K} 4c_i(1 - c_i), \quad (14)$$

where K is the number of ordinal categories considered and c_i is the cumulative proportion of values inside a sample (here, either a singleton or a neighbourhood) of category i or below.
